# Chemically Bonding of Amantadine with Gardenamide A Enhances the Neuroprotective Effects against Corticosterone-Induced Insults in PC12 Cells

**DOI:** 10.3390/ijms160922795

**Published:** 2015-09-21

**Authors:** Jiaqiang Zhao, Lizhi Peng, Wenhua Zheng, Rikang Wang, Lei Zhang, Jian Yang, Heru Chen

**Affiliations:** 1 Institute of Traditional Chinese Medicine and Natural Products, College of Pharmacy, Jinan University, Guangzhou 510632, China; E-Mails: jqzhaodzs@163.com (J.Z.); 15900088863@163.com (L.P.); 13247377339@163.com (L.Z.); 13187048948@163.com (J.Y.); 2 Faculty of Health Sciences, University of Macao, Taipa, Macao, China; 3 National Pharmaceutical Engineering Center for Solid Preparation in Chinese Herbal Medicine, Jiangxi University of Traditional Chinese Medicine, Nanchang 330006, China; E-Mail: wrk168ok@163.com; 4 Guangdong Province Key Laboratory of Pharmacodynamic Constituents of TCM and New Drugs Research, Guangzhou 510632, China

**Keywords:** gardenamide A, amantadine, neuroprotection, nNOS, eNOS, NMDA

## Abstract

Two amantadine (ATD)-gardenamide A (GA) ligands have been designed and synthesized. The bonding of ATD with GA through a methylene carbonyl brigde (**L1**) enhances the neuroprotective effect against corticosterone (CORT)-induced impairments in PC12 cells; while the bonding through a succinyl brigde (**L2**) does not. **L1** reduces the level of reactive oxygen species (ROS) and cell apoptosis generated by CORT. It restores CORT-changed cell morphology to a state that is closed to normal PC12 cells. One mechanism of **L1** to attenuate CORT-induced cell apoptosis is through the adjustment of both caspase-3 and Bcl-2 proteins. Like GA, both nNOS and eNOS might be involved in the neuroprotective mechanism of **L1**. All the evidences suggest that **L1** may be a potential agent to treat depression.

## 1. Introduction

Gardenamide A (GA) was firstly isolated from the fruit of *Gardenia jasminoides* [1], and later was also found in *Rothmannia urcelliformis* [2], which is a widespread small tree in the forests of East Africa. Previously, our research group has developed a scheme to synthesize GA, and found that GA displayed very good neuroprotective effects to serum deprived- and 6-hydroxydopamine (6-OHDA)- induced impairments in PC12 cells [3]. GA was confirmed as the activators of both neuronal nitric oxide synthase (nNOS) and endothelial nitric oxide synthase (eNOS).

As an ongoing project, we are concerned about the problem of depression. It is well known that corticosterone (CORT) is an important steroid hormone in regulating the metabolism of fat, protein, and glucose in various tissues. High concentrations of glucocorticoids showed adverse effects on the central nervous system (CNS), especially on the hippocampus [4,5]. As a stress hormone, repeated injections of CORT decreased the number of reelin+ cells in the subgranular zone (SGZ) of the adult dentate gyrus in a preclinical animal model of depression [6,7]. This evidence indicated that the decreased SGZ reelin expression could bring about a deficit in granule cell maturation, which could be an important event in the pathophysiology of depression. Interestingly, investigations have shown that hippocampal nNOS mediates the depressogenic effects of chronic stress by down-regulating glucocorticoid receptors and suppressing hippocampal neurogenesis [8,9]. It was also indicated that reelin+ cells within the dentate gyrus contain glucorticoid receptors [10]. Therefore, GA, as an effective nNOS activator, is hypothesized as an effective agent to attenuate CORT-induced impairments.

Preliminary study showed that both GA and amantadine (ATD) provide a mild neuroprotective effect against CORT-induced insults in PC12 cells. ATD, which is a weak NMDA receptor antagonist, is currently applied as an antiviral and an anti-PD drug [11]. The mechanism of ATD in treating nervous system disorders is increasingly confirmed by the inhibition of NMDA responses [12]. It has also been disclosed that the binding of ATD with NMDA accelerates channel closure. This channel regulation is of profound physiological significance because it is responsible for the powerful voltage dependence of postsynaptic Ca^2+^ influx at excitatory synapses [13]. Another fact is that CORT markedly facilitates Ca^2+^ influx into the hippocampal neuron leading to neurotoxicity [14]. All these facts taken together suggest that ATD may enhance the neuroprotection of GA against CORT-induced insults. Therefore, in the current investigation, the design and synthesis of two ATD-GA ligands, together with their biology, will be described.

## 2. Results and Discussion

### 2.1. Chemistry

**Scheme 1:**
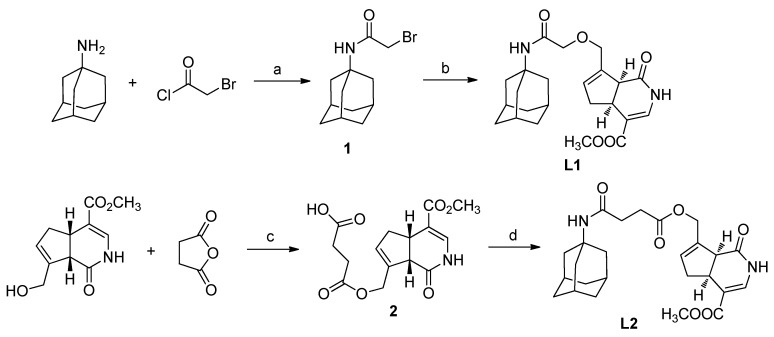
Synthesis of **L1** and **L2**. Reagents and conditions: (a) Et_3_N/DCM, 0 °C → r.t., 68.5%; (b) GA, *n*-BuLi, THF, r.t., 37.6%; (c) Pyridine, r.t., 89.0%; (d) EDCI/HOBt/DIPEA, THF, r.t., 40.6%.

As outlined in Scheme 1, in the current study, there are two bonding modes between ATD and GA. In the first mode, ATD reacted with α-bromoacetyl chloride to form *N*-α-bromoacetyl amantadine (**1**). The yield was 68.5%. *N*-α-chloroacetyl amantadine was also found as one of the side products. Compound **1** then reacted with GA under the interaction of *n*-butyl lithium (*n*-BuLi) at room temperature to give **L1**. The yield was only 37.6%. This reaction belongs to Williamson ether synthesis. Replacement of *n*-BuLi with NaH did not make the reaction work. 

In the second bonding mode, GA firstly reacted with succinic anhydride at room temperature resulted in *O*-β-carboxylpropionyl gardenamide A (**2**) in high yield, which was 89.0%. Then compound **2** coupled with ATD using 1-ethyl-3-(3-dimethylaminopropyl) carbodiimide hydrochloride (EDC·HCl) offered **L2**. The yield was 40.6%.

### 2.2. **L1** Showed the Most Active Neuroprotective Effect

**Figure 1:**
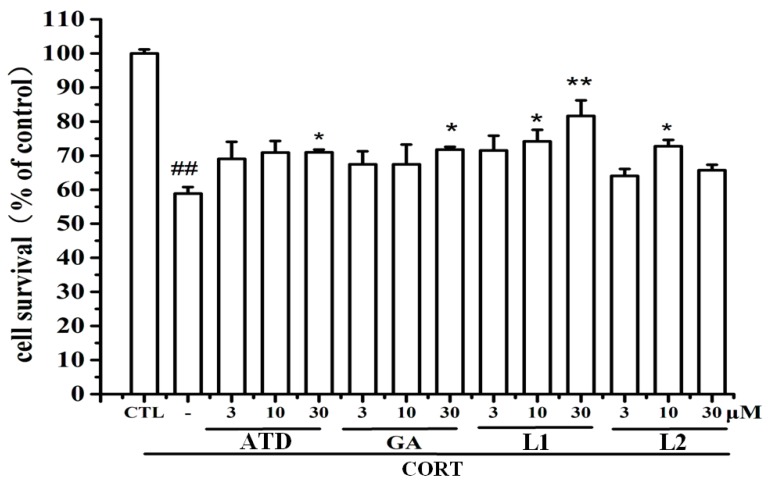
Neuroprotection of amantadine (ATD), gardenamide A (GA), **L1** and **L2** against CORT-induced toxicity in PC12 cells. PC12 cells were treated with ATD, GA, **L1** and **L2** (at a dose of 3, 10 and 30 μM), respectively for 2 h and then incubated with 800 μM CORT for another 24 h. Cells viability was determined by MTT assay. ^##^ *p* < 0.01 *vs.* control group; * *p* < 0.05, ** *p* < 0.01 *vs.* CORT group (*n* = 6).

By using MTT assay, the neuroprotective effect against CORT-induced insult in PC12 cells was evaluated. As indicated in Figure 1, all the tested compounds showed the effect to attenuate CORT impairment. At the dose of 30 µM, **L1** displayed the most active effect if compared to either ATD or GA, even to the combination of ATD and GA (data not shown). As expected, when ATD bonded to GA in the first mode, it enhanced the neuroprotective effect against CORT impairment in PC12 cells. However, **L2** did not show the enhancing effect.

From a view point of chemical structure, in **L1**, ATD bonded to GA in a more compacted and stable mode; in **L2**, ATD bonded to GA in a more spacious and non-stable mode, where the ester bond is easily hydrolyzed. It is likely that the compacted stable array between ATD and GA enhance the binding affinity to nNOS, eNOS, or NMDA.

### 2.3. **L1** Dose-Dependently Attenuated the Increase of ROS Caused by CORT

**Figure 2:**
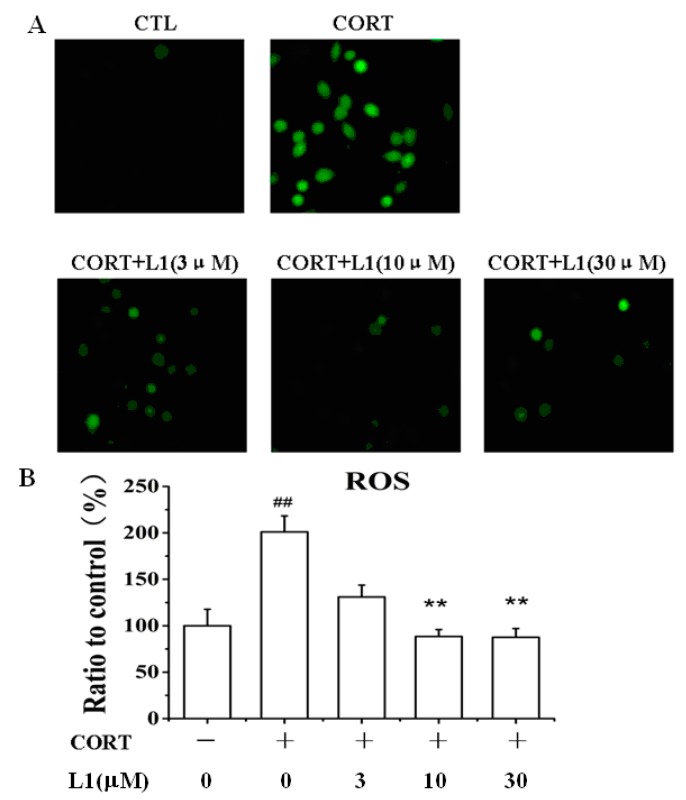
**L1** inhibited CORT-induced ROS production. (**A**) Representative images taken by a fluorescence microscope. The images shown were representative of three experiments; (**B**) Histogram showing the ROS level in PC12 cells. PC12 cells were pretreated with or without **L1** for 2 h (at dose of 3, 10 and 30 μM, respectively), and then treated with or without 800 μM CORT for 24 h. ** *p* < 0.01 *vs.* control group; ^##^ *p* < 0.01 *vs.* CORT-treated group (*n* = 6).

CORT is a stress hormone. Overexposure to CORT may induces an oxidative and inflammatory status in the brain accompanied by decreased antioxidant defenses, lipid peroxidation, DNA damage, mitochondrial dysfunction, and abnormalities in the monoaminergic systems. This status also reduced neurogenesis and neuronal plasticity [15,16]. Interestingly, **L1** dose-dependently attenuated the CORT-induced generation of reactive oxygen species (ROS) (Figure 2A,B). At the dose of 30 µM, **L1** lowered down ROS level from 200% ± 2.1% to 90% ± 1.8%. This function may attribute to GA scaffold in the structure, which was ever reported as an antioxidant against ROS increase caused by serum deprivation [17]. The evidence implies that the incorporation of ATD into GA by the first bonding mode does not affect its activity as antioxidant.

### 2.4. **L1** Reduced CORT-Induced Cell Morphological Change and Apoptosis

**Figure 3:**
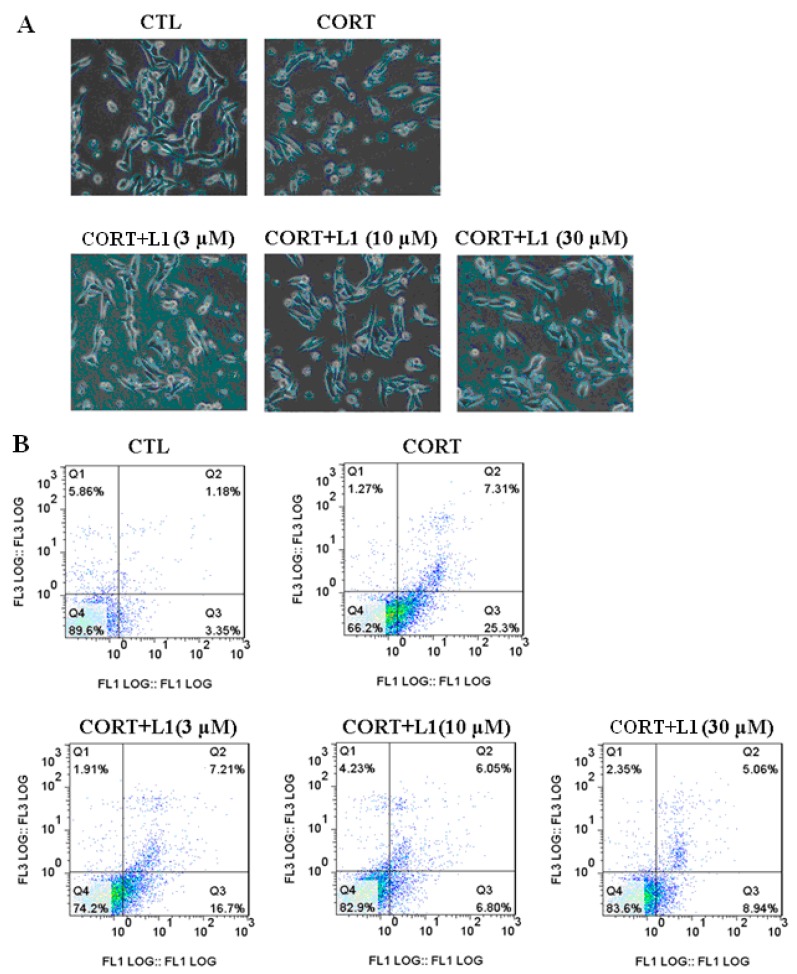
**L1** inhibited CORT-induced morphologic changes and apoptosis in PC12 cells. (**A**) **L1** restored CORT-induced morphologic changes. Representative images were taken by a fluorescence microscope. PC12 cells were pretreated with or without **L1** for 2 h (at dose of 3, 10 and 30 μM, respectively) and then treated with or without 800 μM CORT for 24 h. Cell morphology was significantly damaged by CORT exposure, which was markedly attenuated by F1. The images shown were representative of three experiments; (**B**) Representative scatter diagrams. *X*-axis: The intensity of Annexin V-PE; *Y*-axis: The intensity of Annexin V-PE. PC12 cells were pre-treated with **L1** for 2 h (at dose of 3, 10 and 30 μM, respectively) before the treatment of 800 μM CORT for another 24 h. Cells were stained with Annexin-V and PI. The apoptosis of PC12 was detected by flow cytometry.

In brain, ROS generated by glucocorticoid exposure will finally induce apoptotic neuronal cell death, which appears to be a key mechanism for the development of depression [15,18]. In the current study, treatment of CORT (800 µM for 24 h) increased ROS level (Figure 2A). This intracellular ROS at last resulted in changing cell morphological and apoptosis (Figure 3A,B). It was clearly shown in the first row of Figure 3A that after CORT treatment (800 µM for 24 h), the number of cells and neurite decreased; the cells became small, condensed, and smooth; the ability of cellular adherent was weakened. Fortunately, **L1** dose-dependently restored the cell morphology to the state that is close to the controlled group (Figure 3A, second row). These morphological changes were indicative of cell apoptosis.

As shown in the first row of Figure 3B, basal apoptosis amounted to 1.18%, CORT treatment (800 µM for 24 h) increased apoptosis to 7.31%. **L1** reduced the CORT-induced apoptosis to 5.06%.

It is recognized that ATD could alleviate the dopaminergic neuronal apoptosis following traumatic brain injury (TBI) in the substantia nigra (SN) [19]; while GA displayed the activity to attenuate apoptosis induced by serum deprivation in PC12 cells [17]. Therefore, it is reasonable to deduce that the function of **L1** to reduce CORT-induced apoptosis in PC12 cells may ascribe to both ATD and GA scaffolds within the molecule.

### 2.5. **L1** Increased Caspase-3 (35 kDa) Level and Decreased Cleaved Caspase-3 (19 kDa) Level

**Figure 4:**
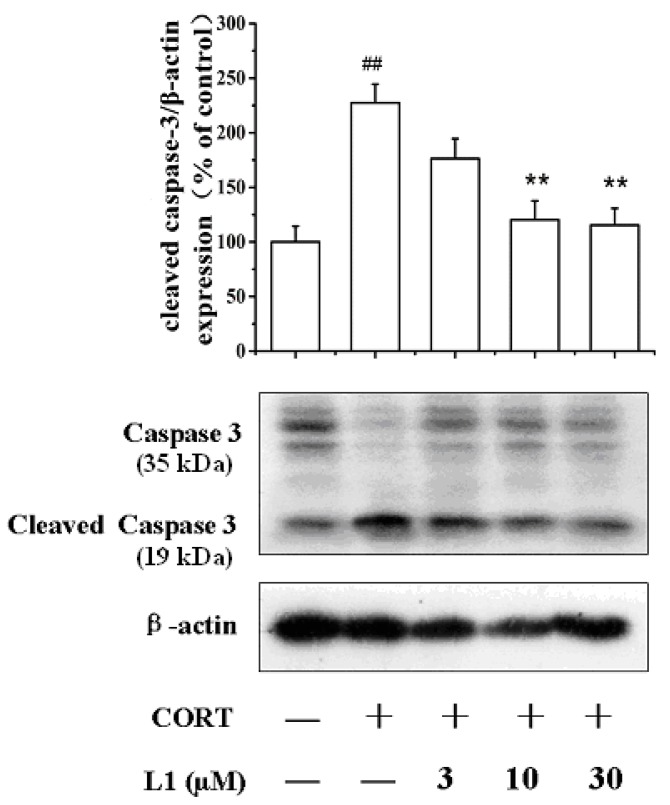
Effect of **L1** on caspase-3 (35 kDa) and cleaved caspase-3 (19 kDa). kDa: kilodaltons. PC12 cells were treated with **L1** (10 μM) for 2 h before exposed to CORT (800 µM) for 24 h. The levels of caspase-3 and cleaved caspase-3 expression were measured using Western Blot. The density of each lane was presented as mean ± standard deviation for at least three individual experiments. ^##^ *p* < 0.01 *vs.* control group; ** *p* < 0.01 *vs.* CORT pretreated group. Blots were quantified using Image J software.

In order to disclose how **L1** attenuate CORT-induced apoptosis, we examined the expression level of caspase-3 and cleaved caspase-3 by Western Blot. We found that CORT decreased the level of caspase-3 (35 kDa, kDa: kilodaltons), while in the mean time increased the expression of cleaved caspase-3 (19 kDa). However, **L1** reversed the CORT-induced effects to both caspase-3 and cleaved caspase-3 (Figure 4). No doubt, caspase-3 was involved in the apoptotic process, where it is responsible for chromatin condensation and DNA fragmentation [20]. The fact is that CORT treatment made caspase-3 cleave into 19 and 16 kDa subunits, which induced cell apoptosis; While **L1** inhibited this process.

### 2.6. **L1** Restored CORT-Inhibited Bcl-2 Expression

**Figure 5:**
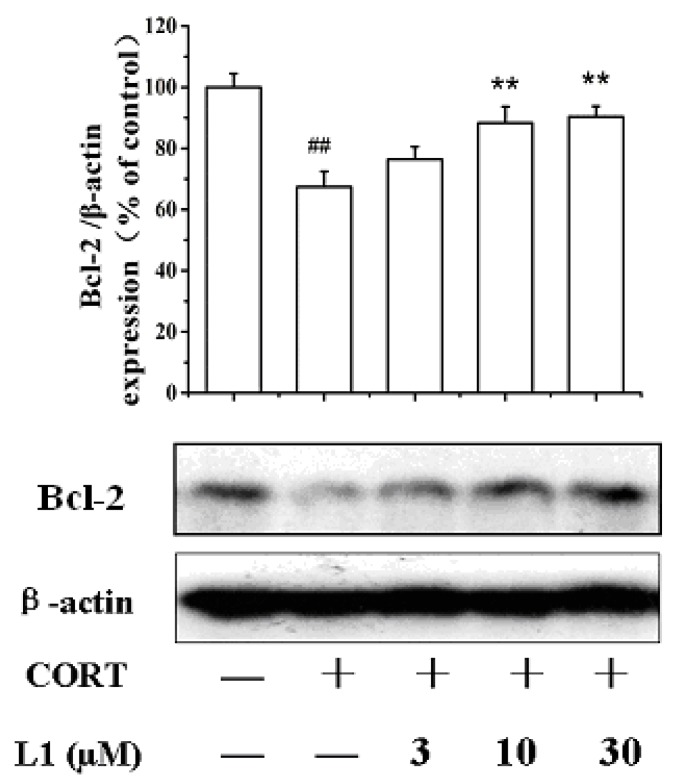
Effect of **L1** on Bcl-2. PC12 cells were treated with **L1** (10 μM) for 2 h before exposed to CORT (800 µM) for 24 h. The expression level of Bcl-2 was measured using Western Blot. The density of each lane was presented as mean ± standard deviation for at least three individual experiments. ^##^ *p* < 0.01 *vs* control group; ** *p* < 0.01 *vs* CORT pretreated group. Blots were quantified using Image J software.

Another apoptosis-related protein is the B-cell lymphona 2 (Bcl-2). Bcl-2 is specifically considered as an important anti-apoptotic protein and is thus classified as an oncogene [21,22]. As shown in Figure 5, CORT treatment (800 µM for 24 h) lowered down Bcl-2 expression. However, **L1** recovered the CORT-inhibited Bcl-2 level. As far as we know, no reports have been displayed that either ATD or GA can activate the expression of Bcl-2. Whether this is the synergic effect between ATD and GA requires further investigation.

### 2.7. nNOS and eNOS might Involve in the Neuroprotection of **L1**

**Figure 6:**
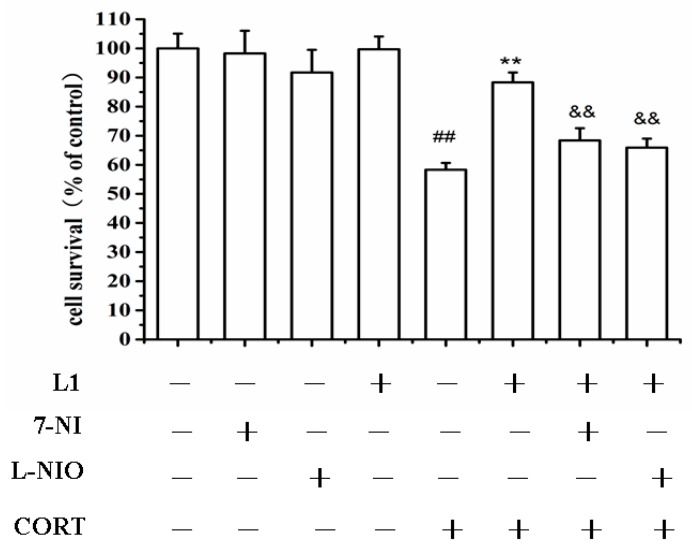
Neuroprotection of **L1** was inhibited by 7-NI and l-NIO. PC12 cells were pretreated with 7-NI (50 μM), and l-NIO (100 μM) for 30 min before the treatment of **L1** (30 µM) for 2 h, and then incubated with CORT (800 μM) for another 24 h. 7-NI: 7-nitroindazole; l-NIO: *N*5-(1-imino-3-butenyl)-l-ornithine. Cells viability were determined by MTT assay. ^##^ *p* < 0.01 *vs.* control group; ** *p* < 0.01, ^&&^ *p* < 0.01, *vs.* CORT group (*n* = 6).

As reported previously, GA is the activators of both nNOS and eNOS [3]. In the present investigation, it was shown in Figure 6 that both nNOS and eNOS were involved in the neuroprotective effect of **L1** against CORT insults. This was indicated by the fact that either the nNOS specific inhibitor 7-nitroindazole (7-NI), or the eNOS specific inhibitor *N*5-(1-Imino-3-butenyl)-l-ornithine (l-NIO), inhibited the neuroprotective effect of **L1** against CORT-induced insults. Therefore, it is likely that both nNOS and eNOS might be involved in the neuroprotective effect of **L1**. The bonding of ATD with GA in the first mode makes **L1** still have the similar functions as GA.

In depressed patients, hippocampal NOS expression is significantly increased [23]. Interestingly, it was found that the non-selective NOS inhibitor, L-*N*^G^-nitroarginine methyl ester (L-NAME), and the nNOS selective inhibitor 7-NI, induce dose-dependent antidepressant-like effects in the forced swimming test [24,25,26]. Another report showed that the clinical antidepressant Fluoxetine raises brain eNOS expression in the arginine depressive rat model [27].

Based on the fact that both nNOS and eNOS might be involved in the neuroprotective mechanism of **L1**, it is likely that **L1** may take effect on depression. However, what the exact effects are requires further investigation.

## 3. Experimental Section

### 3.1. Materials and Methods

Genipin was purchased from Chenddu Kangbang Biotechnology Ltd., Chengdu, China; GA was prepared according to our previous report [3]; Amantadine, EDCI were purchased from Aladdin Reagents Ltd, Shanghai, China; Silica gel for column chromatography was purchased from Qingdao Marine Chemicals Inc., Qingdao, China; Differentiated PC12 cells were purchased from Centre of Cells Resource, Shanghai Institute of Life Science, Chinese Academy of Sciences, China. A014 Typed Nitric Oxide Synthase (NOS) Detection Kit was purchased from Nanjing Jiancheng Institute of Bioengineering; Corticosterone (CORT), 3-(4,5-Dimethylthiazol-2-yl)-2,5-diphenyl tetrazolium bromide(MTT), 2,7-dichlorodihydrofluorescin diacetate (DCFH-DA) and DMSO were purchased from Sigma (St. Louis, MO, USA); Trizol from Tiangen Life Sciences, Beijing, China; 7-Nitroindazole and 1400-W from Enzo (New York, NY, USA); L-NIO from Santa Cluz (Dallas, TX, USA); Fetal bovine serum (FBS) and high-glucose Dulbecco’s modified Eagle’s medium (DMEM), and horse serum were from GIBCO-BRL (Grand Island, NY, USA); BCA protein assay kit was from the Beyotime Institute of Biotechnology (Haimen, China); Annexin V-FITC/PI Apoptosis Detection Kit was from KeyGen (Nanjing, China); Anti-caspase-3 antibody, anti-cleaved caspase-3 antibody, anti-Bcl-2 antibody, and anti-β-actin antibody were purchased from Cell Signaling Technology (Woburn, MA, USA).

NMR spectra were recorded on Bruker AV-300 (Bruker Biospin, Fällanden, Swiss); TMS was used as internal standard. ESI-MS were recorded on Finnigan LCQ Advantage MAX mass spectrometer. HPLC was performed on a LC-100 liquid chromatograph equipped with a tunable LC-100 UV detector (Shanghai Wufeng Inc., Shanghai, China). Pre-coated thin-layer chromatography (TLC) plates (Institute of Yantai Chemical Industry, Yantai, China) were used for TLC. Spots on TLC plates were detected by either a ZF-7A portable UV detector or spraying KMnO_4_ solution followed subsequent heating. *N*,*N*-dimethylformamide (DMF) was refluxed over CaH_2_ for 2 h and redistilled with reduced pressure; Tetrahydrofuran (THF) was dried over sodium thread and then freshly distilled. Dichloromethane (DCM) was dried over P_2_O_5_ for 2 h and redistilled.

### 3.2. Synthesis of N-α-Bromoacetyl Amantadine (**1**)

Amantadine (151.4 mg, 1.0 mmol) was dissolved in 5 ml of dried dichloromethane (DCM) following the addition of Et_3_N (0.17 mL, 1.2 mmol). The solution was cooled down with ice-bath. Then, α-bromoacetyl chloride (0.1 mL, 1.2 mmol) was added via syringe under stirring. The mixture was stirred for 30 min at 0 °C following another 3 h at room temperature. The reaction was terminated by the addition of 20 mL saturated NaHCO_3_ solution, in which the pH value was 9.5. The mixed solution was extracted with DCM three times (3 × 50 mL). All the extracts were combine, and dried over anhydrous MgSO_4_. After filtration, excess solvent was removed by rotatory evaporation under reduced pressure. The residues were then purified by RP-HPLC (Eluent: methanol/H_2_O = 70/30, *V*/*V*) to afford **1** in white solid (198.5 mg, 68.5%). ^1^H NMR (300 MHz, CDCl_3_) δ: 3.93 (s, 2H), 2.10 (m, 9H), 2.03–2.02 (m, 6H); ^13^C NMR (75 MHz, CDCl_3_) δ: 172.9, 42.9, 41.2 (3), 36.2 (3), 29.3 (4); *ESI*-MS (*m*/*z*): Calcd for C_12_H_1__9_BrNO 272.2, found 272.1 [M + H]^+^.

### 3.3. Synthesis of O-(2-(Adamantan-1-ylamino)-2-oxoethyl)gardenamide A **(L1)**

Gardenamide A (112.5 mg, 0.5 mmol) was dissolved in 5 mL fresh distilled THF. The solution was cooled down to 0 °C with ice-bath. Then, *n*-butyl lithium (65.2 mg, 1.0 mmol) was added. The mixture was stirred for 1 h. Afterwards, solution of compound **1** (138.2 mg, 0.5 mmol) in 2 mL fresh distilled THF was added dropwise. The reaction was held at room temperature for another 3 h. 20 mL of water was added to quench the reaction. The mixture was quickly extracted with DCM for 3 times (3 × 50 mL). All the extracts were combine, and dried over anhydrous MgSO_4_. After filtration, excess solvent was removed by rotatory evaporation under reduced pressure. The residues were then purified by RP-HPLC (Eluent: methanol/H_2_O = 65/35, *V*/*V*) to afford **L1** in slight yellow oil (88.2 mg, 37.6%). Purity: 97.3%. ^1^H NMR (300 MHz, CDCl_3_) δ: 7.21 (s, 1H), 5.83 (s, 1H), 4.34–4.14 (q, *J* = 9.6 Hz, 2H), 4.03 (m, 2H), 3.78 (s, 3H, –OCH_3_), 2.97–2.83 (m, 1H), 2.32–2.23 (m, 1H), 2.04 (m, 3H), 1.96–1.94 (d, *J* = 6.0 Hz, 6H), 1.64 (d, *J* = 3.5 Hz, 6H); ^13^C NMR (75 MHz, CDCl_3_) δ:171.0, 166.7, 165.6, 140.8, 137.8, 129.7, 111.4, 60.9, 52.5, 51.7, 51.1, 50.1, 41.5 (3), 40.0, 37.5, 36.2 (3), 29.4 (3); *ESI*-MS (*m*/*z*): Calcd for C_23_H_3__1_N_2_O_5_ 415.2, found 415.3 [M + H]^+^; HR-MS (*m*/*z*): Calcd for C_23_H_3__1_N_2_O_5_ 415.2233, found 415.2235 [M + H]^+^.

### 3.4. Synthesis of O-β-Carboxylpropionyl Gardenamide A **(2)**

Gardenamide A (223.2 mg, 1.0 mmol) was dissolved in 5 mL dried pyridine. Succinic anhydride (200.1 mg, 2.0 mmol) was added. The mixture was stirred at room temperature for 24 h. Removal of pyridine was carried on by freeze drying. The residues were purified by RP-HPLC (Eluent: methanol/H_2_O = 50/50, *V*/*V*) to afford **2** in yellow oil (287.5 mg, 89.0%). ^1^H NMR (300 MHz, CDCl_3_) δ: 7.19 (d, *J* = 3.9 Hz, 1H), 5.85 (s, 1H), 4.40–4.30 (q, *J* = 9.6 Hz, 2H), 3.76 (s, 3H, –OCH_3_), 3.68–3.52 (m, 2H), 2.97–2.91 (dd, *J* = 8.1, 8.1 Hz, 1H), 2.65–2.63 (m, 4H), 2.28–2.22 (dd, *J* = 6.8, 6.8 Hz, 1H); ^13^C NMR (75 MHz, CDCl_3_) δ: 173.4, 172.5, 171.4, 167.5, 142.2, 133.9, 126.7, 109.4, 60.4, 50.6, 39.8, 39.6, 37.3, 36.9, 29.4; *ESI*-MS (*m*/*z*): Calcd for NaC_15_H_17_NO_7_ 346.3, found 346.3 [M + Na]^+^.

### 3.5. Synthesis of O-(4-(Adamantan-1-ylamino)succinyl Gardenamide A **(L2)**

Compound **2** (79.5 mg, 0.25 mmol) was dissolved in 2 mL dried DCM and 2 mL dried DMF. The solution was cooled with an ice-bath. EDCI (72.3 mg, 0.38 mmol) and HOBt (51.3 mg, 0.38 mmol) were added and stirred for 15 min. After addition of DIPEA (0.09 mL, 0.38 mmol) via syringe, ATD (38.2 mg, 0.25 mmol) was added. The mixture was lasted at room temperature for 23 h. Then 20 mL of water was added to quench the reaction. The mixture was extracted with DCM three times (3 × 50 mL). All the extracts were combine, and dried over anhydrous MgSO_4_. After filtration, excess solvent was removed by rotatory evaporation under reduced pressure. The residues were then purified by RP-HPLC (Eluent: methanol/H_2_O = 70/30, *V*/*V*) to afford **L2** in slight yellow oil (44.5 mg, 40.6%). Purity: 98.2%. ^1^H NMR (300 MHz, CDCl_3_) δ: 8.21–8.19 (s, 1H, –NH), 7.19 (s, 1H), 5.87 (s, 1H), 4.95 (m, 2H), 3.73 (s, 3H, –OCH_3_), 3.57–3.55 (m, 2H), 2.90–2.87 (m, 1H), 2.67–2.62 (m, 2H), 2.42–2.37 (m, 2H), 2.19–2.17 (m, 1H), 2.04–2.01 (m, 3H), 1.96–1.95 (m, 6H), 1.66–1.64 (m, 6H); ^13^C NMR (75 MHz, CDCl_3_) δ: 172.4, 170.6, 170.4, 167.0, 136.8, 133.1, 133.0, 62.9, 51.9, 52.0, 48.0, 41.6 (3), 40.1, 36.3 (3), 32.0, 29.6, 29.4 (3); *ESI*-MS (*m*/*z*): Calcd for C_25_H_33_N_2_O_6_ 457.2, found 457.3 [M + H]^+^; HR-MS (*m*/*z*): Calcd for C_25_H_33_N_2_O_6_ 457.2339, found 457.2342 [M + H]^+^.

### 3.6. Culture of PC12 Cells

Differentiated PC12 cells were cultured in high-glucose Dulbecco’s modified Eagle’s medium (DMEM) containing 5% fetal bovine serum (FBS) in 5% CO_2_ and a humidified atmosphere of 95% at 37 °C. The cells were passaged by trypsinization every two to three days. PC12 cells were cultured overnight before use. After washing with DMEM, the cells were plated on 96-well cell culture plate pre-coated with poly-d-lysine and fed with DMEM medium supplemented with 1% FBS, 100 U/mL of penicillin, and 100 µg/mL of streptomycin in a humidified atmosphere of 95% and 5% CO_2_ at 37 °C to avoid over cell growth.

### 3.7. Neuroprotective Activity Tests

Differentiated PC12 cells were plated on 96-well cell culture plate. The culture medium was replaced with serum-free medium. Cells in the control group were cultured in the medium with 1% FBS. Cells in the model group were treated with CORT at 800 µM; Cells in the other groups were treated with ATD, GA, **L1**, and **L2** at different doses (3, 10, 30 μM), respectively, for 2 h before the treatment of CORT (800 µM) for 24 h. Each experiment was repeated at least three times. After 24 h treatment, the medium was removed and replaced with 90 µL of fresh DMEM. Cell viability was determined by MTT assay as described below.

### 3.8. MTT Assay

Cell viability, virtually the mitochondrial activity of living cells, was measured by a quantitative colorimetric assay with 3-(4,5-Dimethylthiazol-2-yl)-2,5-diphenyl- tetrazolium bromide (MTT). After 24 h treatment for the control and the drug-treated groups, the culture medium were removed and replaced with 90 µL of fresh DMEM. An aliquot (10 µL) of 5 mg/mL MTT in phosphate-buffered saline (PBS) was added to each well. A well added 10 µL of 5 mg/mL MTT and 90 µL DMEM was set as negative control. The plates were then incubated at 37 °C for 4 h, then supernatants were discarded. 100 µL of DMSO solutions were added to each wells and the solutions were mixed thoroughly. Then the plates were incubated at 37 °C for another 10 min. Each sample was mixed again and the resultant formazan was measured by its absorbance at 570 nm using a BIO-RAD680 plate reader (Thermo, Walsam, MA, USA). The experiments were repeated at least 3 times and compared with the control experiment.

### 3.9. Neuroprotective Inhibition Test

Differentiated PC12 cells were pretreated with 7-NI (50 μM) and L-NIO (100 μM), for half an hour respectively. Then the cells were treated with L1 (30 µM) for 2 h before the addition of CORT at a final concentration of 800 μM. The cells were cultured for another 24 h. The cell viability was tested by MTT assay as described above.

### 3.10. Measurement of Intracellular ROS 

ROS generation was evaluated using 2,7-dichlorodihydrofluorescein diacetate (DCFH-DA, Sigma-Aldrich), a radical sensor and membrane-permeable probe that is de-esterified intracellularly. The dye penetrates cells freely and is then hydrolyzed to DCFH by intracellular esterase. The DCFH is then trapped inside the cells. Upon oxidation by ROS, DCFH yields a highly fluorescent product, the dichlorofluorescein (DCF). Treated cells were loaded with DCFH-DA (50 mM final concentration) in DMEM media in dark for 30 min. The cells were rinsed twice with PBS solution and the fluorescence was analyzed using a high content screening system (ArrayScanVTI, Thermo Fisher Scientific, Pittsburgh, PA, USA). The excitation wavelength was set at 488 nm and the emission wavelength was set at 525 nm.

### 3.11. Morphologic Changes 

PC12 cells grown on 48-well plates were treated with **L1** and/or CORT as described above. After that, the medium was removed by washing with PBS. Morphologic changes were observed by phase-contrast microscopy and cells images were taken using a fluorescence microscope (IX71, Olympus, Tokyo, Japan).

### 3.12. Flow Cytometry Analysis for Cell Apoptosis

The number of viable, early apoptotic, and late apoptotic cells was detected and analyzed by flow cytometry using an annexin V-PE and Propidium Iodide (PI) double staining kit (Biotium, Hayward, CA, USA) in accordance with the manufacturer’s instructions. Briefly, cells were collected by centrifugation and resuspended in 100 μL of 1× binding buffer with 5 μL annexin V-PE and 5 μL PI. After incubation at room temperature for 15 min in the dark, 200 μL of 1× binding buffer was added. The cells were analyzed using a FACScan and CELLQuest software (Biomedika, QC, Canada). At least 20,000 events per assay well were included and represented as dot plots. Annexin+/PI− cells were considered early apoptotic cells, annexin+/PI+ late apoptotic cells, and annexin−/PI− viable cells. Percentage of apoptosis was calculated based on all the Annexin+/PI− (early apoptotic) plus the annexin+/PI+ (late apoptotic) cells.

### 3.13. Western Blot

Cells from different experimental conditions were lysed with ice-cold RIPA lysis buffer as described previously [28]. Protein concentration was measured with a BCA protein assay kit according to the manufacturer’s instructions. Samples with equal amounts of proteins were separated on 8% polyacrylamide gels, then were transferred to PVDF membrane, and probed with selective anti-phospho antibodies against caspase-3 (35 kDa, kDa: kilodaltons), cleaved caspase-3 (Asp175, 19 kDa), and Bcl-2 (C-terminal, 28 kDa), respectively. Blots were subsequently stripped from antibodies and re-probed with the respective pan antibody or anti-β-actin in order to confirm the equal protein loading. Immunoblots shown in the figures correspond to a representative experiment that was repeated four times with similar results.

### 3.14. Data Analysis and Statistics

Statistical analyses were conducted with the software SPSS 13.0 (SPSS Ltd., Chicago, IL, USA). Data are expressed as the mean ± SEM or mean ± SD. Variation between groups was analyzed using one-way ANOVA, which was followed by Student-Newman-Keuls or Dunnett’s T3 procedures when the assumption of equal variances did not hold. *p* Value < 0.05 was considered statistically significant. 

## 4. Conclusions

In summary, two ATD- GA ligands have been designed and synthesized. Chemically bonding of ATD with GA through a methylene carbonyl bridge (**L1**) enhances the neuroprotective effect against CORT-induced insults in PC12 cells; while bonding through succinyl bridge (**L2**) does not. L1 reduces the level of reactive oxygen species (ROS) and cell apoptosis generated by CORT. It restores CORT-changed cell morphology to a state that is closed to normal PC12 cells. One mechanism of L1 to attenuate CORT-induced apoptosis is through the adjustment of both caspase-3 and Bcl-2 proteins. Both nNOS and eNOS might involve in the neuroprotective mechanism of **L1**, which is similar to GA, All the evidences suggest that **L1** might be a potential agent to treat depression.
